# Feedback on Physical Activity Through a Wearable Device Connected to a Mobile Phone App in Patients With Metabolic Syndrome: Pilot Study

**DOI:** 10.2196/13381

**Published:** 2019-06-18

**Authors:** Up Huh, Young Jin Tak, Seunghwan Song, Sung Woon Chung, Sang Min Sung, Chung Won Lee, Miju Bae, Hyo Young Ahn

**Affiliations:** 1 Department of Thoracic and Cardiovascular Surgery Pusan National University Hospital Busan Republic of Korea; 2 Biomedical Research Institute Pusan National University Hospital Busan Republic of Korea; 3 Department of Family Medicine Pusan National University Hospital Busan Republic of Korea; 4 Department of Neurology Pusan National University Hospital Busan Republic of Korea

**Keywords:** electronic activity monitor, wearable devices, metabolic syndrome, physical activity

## Abstract

**Background:**

Little is known of the effect of wearable devices on metabolic impairments in clinical settings. We hypothesized that a wearable device that can monitor and provide feedback on physical activity may help resolve metabolic syndrome.

**Objective:**

This study aimed to examine the objective effects of the use of these devices on metabolic syndrome resolution.

**Methods:**

Patients diagnosed with metabolic syndrome were recruited. Participants were prescribed regular walking using a wearable device (Coffee WALKIE +Dv.3, GC Healthcare CI, Korea) on their wrist for 12 weeks. Participants received self-feedback on the amount of their exercise through an app on their mobile phone. The information on physical activities of the participants was uploaded automatically to a website. Thus, a trained nurse could provide individuals with feedback regarding the physical activity via telephone consultation on alternate weeks. Blood pressure (BP), body composition, fasting plasma glucose, and lipid profiles were recorded. The primary outcome was metabolic syndrome resolution. The secondary outcome was an improvement in the components of metabolic impairment.

**Results:**

Of the 53 participants recruited, 20 participants with a median age of 46 (range 36-50) years completed the trial. There was no significant difference in the amount of calorie expenditure at weeks 4, 8, and 12. After 12 weeks, metabolic syndrome was resolved in 9 of 20 participants (45%), and the mean number of metabolic impairment components per person decreased from 3.4 to 2.9. Particularly, the mean systolic and diastolic BP decreased from mean 136.6 (SD 18.5) mm Hg to mean 127.4 (SD 19.5) mm Hg and from mean 84.0 (SD 8.1) mm Hg to mean 77.4 (SD 14.4) mm Hg (both *P*=.02), respectively.

**Conclusions:**

This study found that a 12-week intervention via feedback, based on a wearable physical activity monitor, helped metabolic syndrome patients to be more engaged in regular walking and it improved impaired metabolic components, especially in BP. However, some practical challenges regarding patients’ adherence and sustained engagement were observed.

## Introduction

Metabolic syndrome is a constellation of cardiovascular disease risk factors, such as abdominal obesity, hyperglycemia, hypertension, and hyperlipidemia [1]. With the increasing prevalence of obesity, metabolic syndrome has been reported as one of the most common health conditions among obese people worldwide [2]. Insufficient physical activity is a major cause of obesity and metabolic syndrome, and even small increases in physical activity can have a significant beneficial impact on metabolic syndrome and the prevention of cardiovascular disease [3]. Exercise is known to enhance insulin sensitivity by increasing the AMP-activated protein kinase activity, promoting translocation of glucose transporter type 4 to the cell membrane, thereby boosting glucose uptake. The decreases in intramuscular saturated fatty acids and stimulated beta cell activity also contribute to attenuating insulin resistance [4]. Despite evidence of improvement in metabolic impairment with regular exercise [5], the number of individuals involved in physical activity remains low, and sedentary lifestyle is prevalent, with less than 25% of people estimated to be engaging in regular physical activity [6].

As activity-tracking devices have become smaller, cheaper, and more readily wearable, it is predicted that they will be used extensively for various purposes [7]. Advances in wearable devices and gathering of personal data provide patients with chronic diseases the potential to engage in self-management. However, the data collected by the wearable devices are rarely integrated into the programming of regimens for impaired metabolic conditions. Moreover, evidence supporting the sustained use of data derived from wearable devices or their positive effects on health outcomes is lacking as most studies have mainly focused on establishing the feasibility of the devices and the association between measured physical activity and short-term benefits [8-10]. Recent research indicates that feedback on activity monitoring can successfully increase physical activity levels and lead to beneficial outcomes in the management of target diseases [11]. However, they focused on patients with diabetes mellitus [12,13], heart failure [14], or chronic pulmonary disease [15]. Little has been demonstrated regarding whether use of wearable devices may be a pragmatic option for metabolic syndrome in a clinical setting.

Mobile phones allow users to track their path by connecting to the internet with apps. Internet-based interventions seem to motivate people to increase physical activity with a relatively low cost, time, and effort [16]. Mostly used throughout the day, mobile phones are considered to be a good tool for tracking physical activity in real time. Electronic activity monitors play a potential role as a delivery medium by replicating most aspects of pedometer-based interventions. These monitors measure physical activity or behavior indicators, such as heart rate, and are connected with a mobile device through an app or personal computer to provide extensive feedback. The feedback can be more individualized than that offered in clinical assessments and can include social comparisons, multiple charts, and markers of progress toward individual goals. Considering the ubiquity of mobile phones, few clinical trials have been conducted to assess the impact of the application of mobile phone-wearable step trackers on improvement in metabolic impairment. A number of previous studies have been designed to explore the effectiveness of mobile phone apps in weight loss [17,18] or in increasing physical activity [19,20]. However, they failed to include people with metabolic syndrome and to incorporate a wearable device into their interventions, and none of them were done in a clinical setting.

Given that many people try to use wearable devices compatible with mobile phones to boost physical activity with little evidence-based practice, it is clinically pressing to demonstrate the potential of feedback via this technology for the management of outpatients with metabolic syndrome. There is also a need to assess whether there is any obstacle when applied to real clinical situations. Therefore, we conducted a pilot study aimed to explore the potential and barriers of the application of these devices for metabolic syndrome management in a clinical setting.

## Methods

### Study Participants

The study included patients diagnosed with metabolic syndrome based on the results of a comprehensive health examination at a health promotion center at Pusan National University Hospital (Busan, South Korea) between March and December 2016. The study was conducted in accordance with the Declaration of Helsinki and was approved by the Institutional Review Board at Pusan National University Hospital (review reference number: E-2015030). All participants consented to the study protocols and the subsequent publication of their respective findings.

Inclusion criteria were as follows: (1) diagnosed with metabolic syndrome by a doctor, (2) aged between 20 and 64 years, (3) possession of a mobile phone and a daily mobile phone user for the past 3 months, and 4) no plans to change medication within 3 months after the start of the study.

### Definition of Metabolic Syndrome

Metabolic syndrome was diagnosed by a family doctor according to the 2009 Joint Interim Statement issued by a number of international organizations and expert groups [21]. The definition requires the presence of three or more of the following five components: (1) central obesity (ie, waist circumference of ≥85 cm for women and ≥90 cm for men according to the Korean Society for the Study of Obesity [22]), (2) hyperglycemia (ie, fasting plasma glucose [FPG] ≥100 mg/dL or the use of antidiabetes medication), (3) high blood pressure (BP) (ie, systolic BP ≥130 mm Hg or diastolic BP ≥85 mm Hg or the use of antihypertensive medication), (4) hypertriglyceridemia (ie, fasting plasma triglycerides ≥150 mg/dL or the use of lipid-lowering medication), and 5) low high-density lipoprotein cholesterol (HDL-C) (ie, fasting plasma HDL-C<50 mg/dL for women, <40 mg/dL for men). Patients with any of the following conditions were excluded: (1) any cancer or uncontrolled metabolic or cardiovascular disease, (2) prescriptions for medication that may affect metabolism, and (3) disability or difficulty with regular walking. All participants were recommended for regular exercise of at least five times per week by a doctor as part of the treatment for metabolic syndrome, according to the current guideline [21].

### Data Collection

All study participants underwent an 8 hour-fasting blood test as part of a comprehensive health examination. Data on medical history and anthropometric measurements of participants were collated. Participants also completed a questionnaire about demographics, medical history (diagnosis of or medication for hypertension, diabetes mellitus, or dyslipidemia), and health-related habits (smoking, drinking, and alcohol). To assess physical activity at baseline, participants were asked about the frequency and duration of vigorous and light/moderate physical activity. Individuals who were classified as inactive reported no sessions of light/moderate or vigorous activity of at least 10 minutes’ duration. Those classified as having some activity reported at least one session of light/moderate or vigorous physical activity of at least 10 minutes’ duration but did not meet the definition of regular exercise. Those classified as having regular activity reported three or more sessions per week of vigorous activity lasting at least 20 minutes or five or more sessions per week of light/moderate activity lasting at least 30 minutes in duration [6,21]. Participants were divided into nonsmokers, former smokers, or current smokers, and into nondrinkers (0-98 g/week) or drinkers defined as drinking of an average of seven cups for men and five or more cups for women, more than two times per week [23].

Participants’ BP was tested three times in the sitting position after a 15-minute rest using a BP-203 RVII (Colin Corp, Aichi, Japan), and the average measurement was recorded. Body weight and height were measured using a digital scale and stadiometer (BSM370, Biospace Co Ltd, Seoul), with patients wearing a light gown without shoes. Body mass index (BMI) was calculated as weight (kg) divided by height squared (m^2^). Waist circumference was assessed by trained examiners (following a normal expiration) at the midpoint between the lower costal margin and the iliac crest, to the nearest millimeter. Body composition, including percentage body fat and muscle mass, were calculated via bioelectric impedance analysis (Inbody 720, Biospace Co Ltd, Seoul). Blood samples were collected from an antecubital vein after an 8-hour fast. Samples were then analyzed at a laboratory in our hospital. Lipid profiles were tested using an autoanalyzer (Hitachi 747, Hitachi Corp, Japan) and an enzymatic colorimetric method. FPG levels were evaluated using a glucose oxidase method and a Synchron LX 20 (Beckman Coulter, Fullerton, CA). All participants were assessed again at 12 weeks after the start of the intervention.

### Intervention Using the Wearable Device

All participants were advised to walk aiming at the consumption of a minimum of 150 kcal per day, which was set based on the standard recommendations for metabolic syndrome encouraging a daily minimum of 30 minutes of moderate-intensity (such as brisk walking) physical activity [21]. To estimate the required amount of physical activity for our metabolic syndrome participants, we referenced the study that determined the effects of exercise amount and intensity of metabolic syndrome [24]. The intervention they used for the exercise training group with a low amount of moderate-intensity physical activity was equivalent to 1221 (SD 222) kcal/week with no significant difference between genders. Considering their participants’ mean BMI of 29.9 (SD 3.2) kg/m^2^, and the estimated mean BMI of 23.1 (SD 0.1) kg/m^2^ in Koreans with metabolic syndrome (referenced to the study on 6561 metabolic patients among a nationally representative sample of Koreans) [22], it made sense to set the calorie goal of 150 kcal per day loss for our participants to consume by exercising. Rather than informing the participants about the number of steps to walk, we had them put the minimum calorie goal of 150 kcal loss on their mobile phone app, which was connected to the wearable device that automatically calculated the required step counts to be taken depending on the weight and height of the participants. Then when the user walked, the app measured the estimated calorie consumed, taking into account the walking speed.

Participants were given a wearable device (Coffee WALKIE +Dv.3, GC Healthcare CI, Korea) fitted on the wrist or waist. This wearable device has been certified by the Korea Testing Laboratory (Certification Number: MSIP-CRM-NSJ-Coffee). The device used was validated on the wrist with 86.7% accuracy for the calories burned and 90.5% for the step counts. Considering previous data showing that the waist attachment site detects step counts better than that on the wrist [25,26], we expected similar accuracy regarding the waist placement of the device in our study. Moreover, increased wear times can occur with the wrist placement site [27] and the US National Health and Nutrition Examination Survey also used a step counter worn on the wrist (2008-2014) or the waist (2003-2006) [25]. Therefore, we permitted the participants to wear the device either on the waist or wrist, according to their preference. However, all participants ultimately chose their nondominant wrist as the attachment site, saying that it would be more comfortable for all-day wear, handling, and checking of the device. Detailed instructions on wearable device usage were provided during face-to-face/one-on-one contact by the study coordinator with each participant. Demonstration and instruction sheets including on how to turn on/off and how to operate the wearable device, download the app, and connect the mobile phone and the wearable device to Bluetooth were performed. Subsequently, the compatible app was installed on the mobile phones of the participants, and this was connected to the wearable device. To ensure that the participants become used to the device, they were asked to individually try using it several times under supervision. This practice lasted for approximately 30 minutes. Each participant also received a log-in identity and password that granted them access to the mobile phone app and website where they could track their physical activity over the 12-week study duration. The number of steps taken and calories consumed were displayed on the device’s screen. In addition, participants could check if they had achieved the amount of daily walking. Their workout records were also registered on an online program. An administrative webpage granted the researchers access to check and track the step counts of the participants on a daily basis.

Participants were instructed to wear the device for as many waking hours as possible except while swimming and bathing. All days of exercise were included in the analysis except when the number of step counts exceeded 25,000 in one day, which is considered an extremely high count, or when participants withdrew consent [28]. If the data from the wearable device were not transmitted to the Web server for three consecutive days, an automatic text message was sent to the participant to encourage the use of the device and to see if they had any issue with the device (in which case, they were asked to contact the researcher to address the issue). We then checked the Web server again to see whether step count data were being detected. If no response was received from the user for 6 days in a row, the researcher called to ask if they had any issue with the device or whether it was being used appropriately. Participants who failed to respond to the researcher and those who rejected participation were considered to be dropouts. Thus, we were able to include step records of at least one day of every week. Then we estimated the daily step counts by dividing the total step counts per week by seven. To be included in the analysis, at least one day with any step counts was required. Laboratory-based studies typically require 4 days or more of valid data to be included as a study sample. However, quite a few studies required at least one valid day to be included in the analysis, a method that is consistent with the original examinations of the National Health and Nutrition Examination Survey physical activity data.

**Figure 1:**
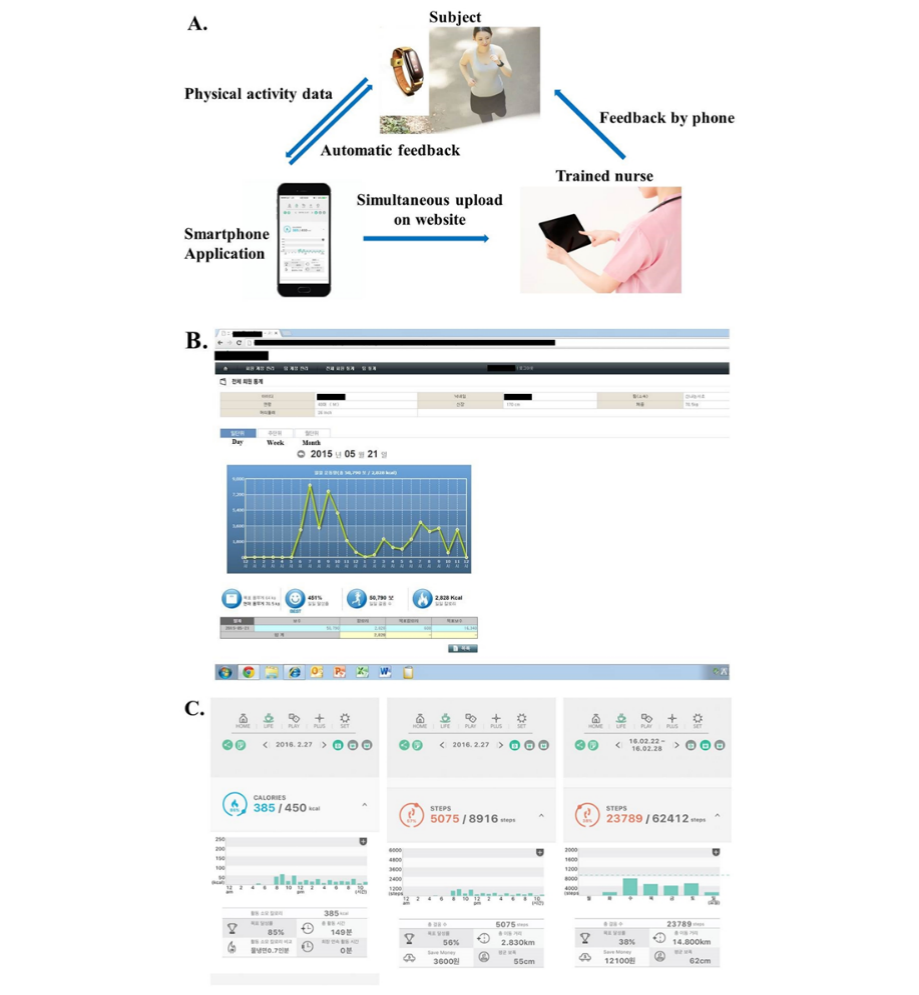
Overview of the intervention using the wearable device and a mobile phone app. (A) Flowchart of participants’ physical activity data collection. (B) Administrative webpage allowing researchers to track the participants’ physical activity. (C) App screen on the participants’ mobile phones presenting the goal setting of physical activity, progress toward the daily goal, total daily steps taken, and estimation of calories burned by physical activity performed.

Over the 12 weeks of the trial, participants were provided with feedback by the nurse via phone on alternate weeks. A trained nurse phoned each participant for personal counseling on their physical activity every other week at an agreed-on time. This included counseling regarding exercise practices and encouragement to continue with the exercise regimen based on current guidelines and recommendations on physical activities. The telephone feedback followed a standardized script (Multimedia Appendix 1) but was flexible depending on the individual, including goal achievement. The consultant answered questions or discussed problems regarding the use of the wearable device, the app (Figure 1), and the exercise, and also provided encouragement to continue with the exercise. This intervention was designed to help improve problem solving, goal setting, and self-monitoring. Participants also had access to additional resources online.

### Statistical Analysis

The sample size for the study was calculated based on the results of a randomized weight-loss intervention using self-monitoring mobile devices [29] that showed an effect size of 0.53. The sample size was 24 for two-sided tests of significance at alpha=.05 and power 1−beta=80%. To account for attrition, we attempted to recruit approximately 30 participants in total.

Data are presented as frequency or proportion and mean and standard deviation (SD) for normally distributed values, or medians and interquartile ranges (IQRs) for nonnormally distributed values. Changes in the components of metabolic syndrome for each participant were examined from baseline to follow-up visit (week 12). The Shapiro-Wilk test was employed to test the normality assumption. To compare baseline characteristics between the study completed and uncompleted groups, we employed the independent *t* test for age and the chi-square test for categorical variables. Comparisons of metabolic components between baseline and at week 12 were conducted using the paired *t* test (waist circumference, body fat mass, and body fat rate). Because some metabolic parameters were nonnormally distributed, the Wilcoxon signed-rank test was used to detect differences between baseline and at week 12 (FPG, systolic/diastolic BP, triglycerides, HDL-C, low-density lipoprotein cholesterol, and total cholesterol). A repeated-measures analysis of variance (ANOVA) was conducted to verify the difference in step count and calorie expenditure changes by walking, at weeks 4, 8, and 12. All analyses were performed using SAS version 9.3 (SAS Institute, Cary, NC, USA), and a *P* value of <.05 was considered statistically significant.

## Results

### Participant Characteristics

**Figure 2:**
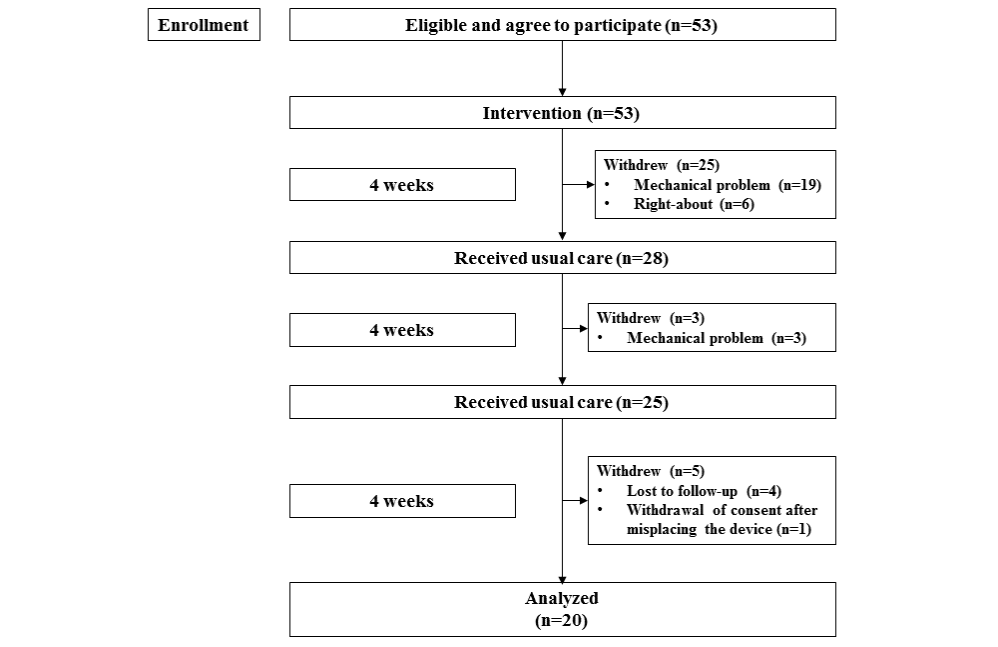
Flowchart of patient enrollment and the reasons for dropout. Right-about: withdrawal of consent within 24 hours; lost to follow-up: participant neither picked up the counseling call nor provided any reasons.

**Figure 3:**
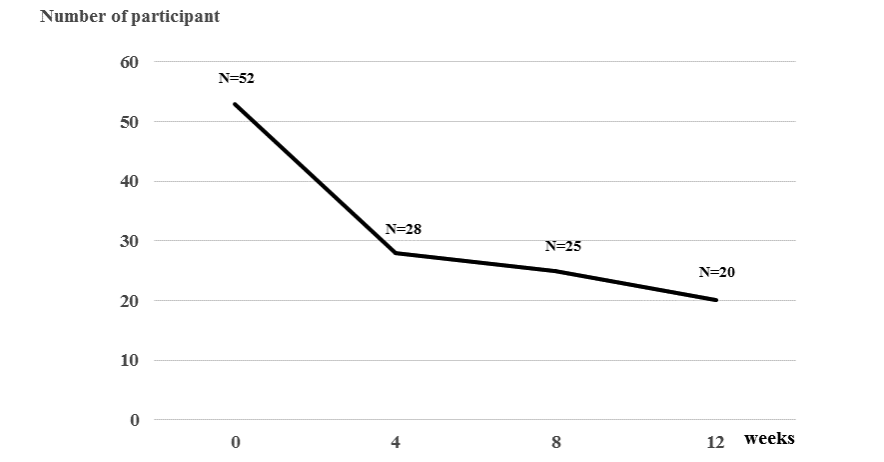
Timeline of participant dropout.

Of the 53 participants recruited, 20 participants with a median age of 46 years completed the exercise monitoring schedule over 12 weeks (Figure 2). The baseline characteristics of the participants who completed the intervention are presented in Table 1. There was a statistically significant difference in participants’ age between those who did and did not complete the intervention (mean 51.97, SD 8.49 years vs mean 44.20, SD 9.55 years; *P*=.003) (Table 1). The majority of participants ceased monitoring their exercise within the first few weeks of the study (Figure 3) primarily due to miscommunication between their mobile device and the wearable device. Only five of the participants who completed the trial (n=20) responded that they had regularly exercised at baseline, whereas 55% (11/20) of them had been physically inactive.

**Table 1:** Clinical characteristics of the study participants.

Variables		Total		Incomplete group	Complete group	*P* value^a^
Participants, n (%)		53 (100)		33 (63.3)	20 (37.7)	—
Age (years), mean (SD)		49.04 (9.60)		51.97 (8.49)	44.20 (9.55)	.003
Male, n (%)		39 (73.6)		22 (66.7)	17 (85)	.20
Current smoker, n (%)		11 (20.8)		5 (15.2)	6 (30)	.17
Former smoker, n (%)		10 (18.9)		6 (18.2)	4 (20)	.30
Alcohol drinker,^b^ n (%)		18 (34.0)		12 (36.4)	6 (30)	.77
Regular exercise,^c^ n (%)		19 (35.8)		14 (42.4)	5 (25)	.20
Physically inactive,^d^ n (%)		29 (54.7)		18 (54.5)	11 (55)	.81
**Medication, n (%)**						
	Antihypertensive	25 (47.2)	16 (48.5)		9 (45)	.81
	Antidiabetic	15 (28.3)	11 (33.3)		4 (20)	.30
	Antihyperlipidemia	18 (34.0)	13 (39.4)		5 (25)	.28
**Total number of abnormal metabolic components, n (%)**						.19
	3	31 (58.5)	18 (54.5)		13 (65)	
	4	13 (24.5)	7 (21.2)		6 (30)	
	5	9 (17.0)	8 (24.2)		1 (5)	
**Have abnormal metabolic component, n (%)^e^**						
	Central obesity	44 (83.0)	26 (78.8)		18 (90)	.26
	Hyperglycemia	29 (54.7)	16 (48.5)		13 (65)	.59
	High blood pressure	48 (90.6)	31 (93.9)		17 (85)	.12
	Hypertriglyceridemia	44 (83.7)	30 (90.9)		14 (70)	.002
	Low high-density lipoprotein cholesterol (HDL-C)	29 (54.7)	16 (48.5)		13 (65)	.24

^a^*P* value was obtained by chi-square test or independent *t* test.

^b^Alcohol drinking was defined as drinking of an average of seven cups for men and five or more cups for women, more than two times per week.

^c^Regular exercise was defined as having regular activity three or more sessions per week of vigorous activity lasting at least 20 minutes, or five or more sessions per week of light/moderate activity lasting at least 30 minutes in duration.

^d^Physically inactive was classified as participation in no sessions of light/moderate or vigorous activity of at least 10 minutes’ duration.

^e^Central obesity: waist circumference ≥90 cm for men, ≥85 cm for women. Hyperglycemia: fasting plasma glucose ≥100 mg/dL or the use of antidiabetes medication. High BP: systolic BP ≥130 or diastolic BP ≥85 mm Hg or the use of antihypertensive medication. Hypertriglyceridemia: fasting plasma triglyceride ≥150 mg/dL or the use of lipid-lowering medication. Low HDL-C: fasting plasma HDL-C<50 mg/dL for women, <40 mg/dL for men.

### Mean Number of Steps and Amount of Calorie Expenditure

The mean number of steps per week on weeks 4, 8, and 12 are described in Table 2. For the first 4 weeks, the participants walked 7616 steps daily and then increased the step counts to 8244, which was equivalent to a 17.5% increase from week 4, for the next 4 months. Between week 8 and week 12, the number of steps remained at the same level as that of week 4. Although there was no statistically significant upward tendency in step counts over time, the results indicated that the participants walked more for the first 8 weeks after enrollment in the trial and maintained the amount of walking at the same level for the last 4 weeks. Thus, it was assumed that the participants consumed a mean of approximately 420 calories daily by walking. Additionally, there was no significant difference in week 4 (*P*=.90), week 8 (*P*=.52), and week 12 (*P*=.63) in step counts between the participants who had regularly exercised before the study and those who had not (Table 2).

### Resolution of the Metabolic Syndrome Status

**Figure 4:**
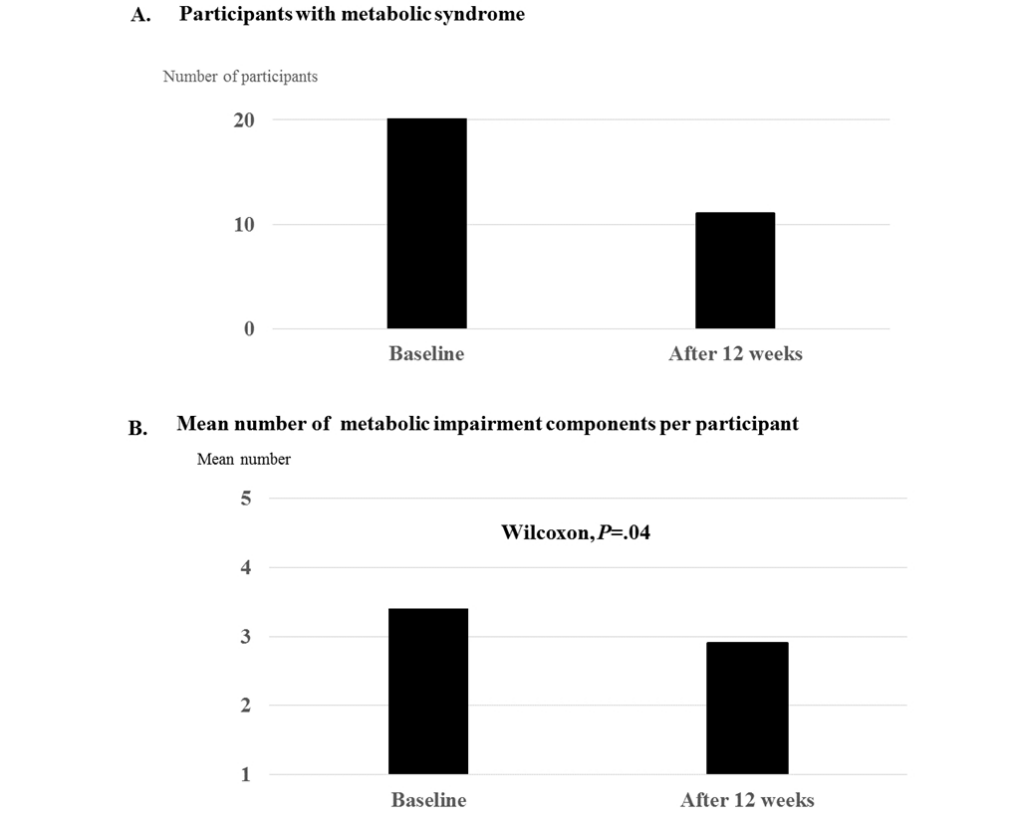
Change in the metabolic abnormality status in study participants from baseline to follow-up at 12 weeks. (A) Resolution of metabolic syndrome. (B) Changes in components of metabolic syndrome.

At 12 weeks following the intervention, metabolic syndrome was resolved in 9 of 20 (45%) participants. We also noted a significant reduction in metabolic syndrome prevalence between baseline and follow-up (Figure 4). The number of metabolic abnormalities decreased in 11 of 20 (55%) participants. In 7 of 20 (35%) participants, there was no change in the number of metabolic abnormalities following the intervention. Metabolic syndrome was aggravated in 1 (5%) participant.

**Table 2:** The daily mean number of steps walked and estimated calories burned (N=20) for groups 1 and 2^a^.

Week and measure		Total (N=20), mean (SD)	Group 1 (n=5), mean (SD)	Group 2 (n=15), mean (SD)	*P* value^b^
**Week 4**					
	Daily steps	7615.8 (3669)	7811.3 (2622)	7550.7 (4036)	.90
	Daily calories burned	410.4 (171)	468.0 (117)	391.2 (184)	.40
**Week 8**					
	Daily steps	8244.4 (3746)	9202.0 (4062)	7925.2 (3727)	.52
	Daily calories burned	445.9 (208)	548.7 (258)	411.7 (187)	.30
**Week 12**					
	Daily steps	7510.4 (3525)	8194.0 (3440)	7282.6 (3640)	.63
	Daily calories burned	413.7 (211)	448.6 (160)	398.2 (172)	.57

^a^Group 1: the participants who regularly exercised at baseline; group 2: the participants who did not regularly exercise at baseline.

^b^Derived from a *t* test to compare step counts and calories burned between the participants who regularly exercised at baseline and those who had not.

#### Mean Change of Each Metabolic Component

**Figure 5:**
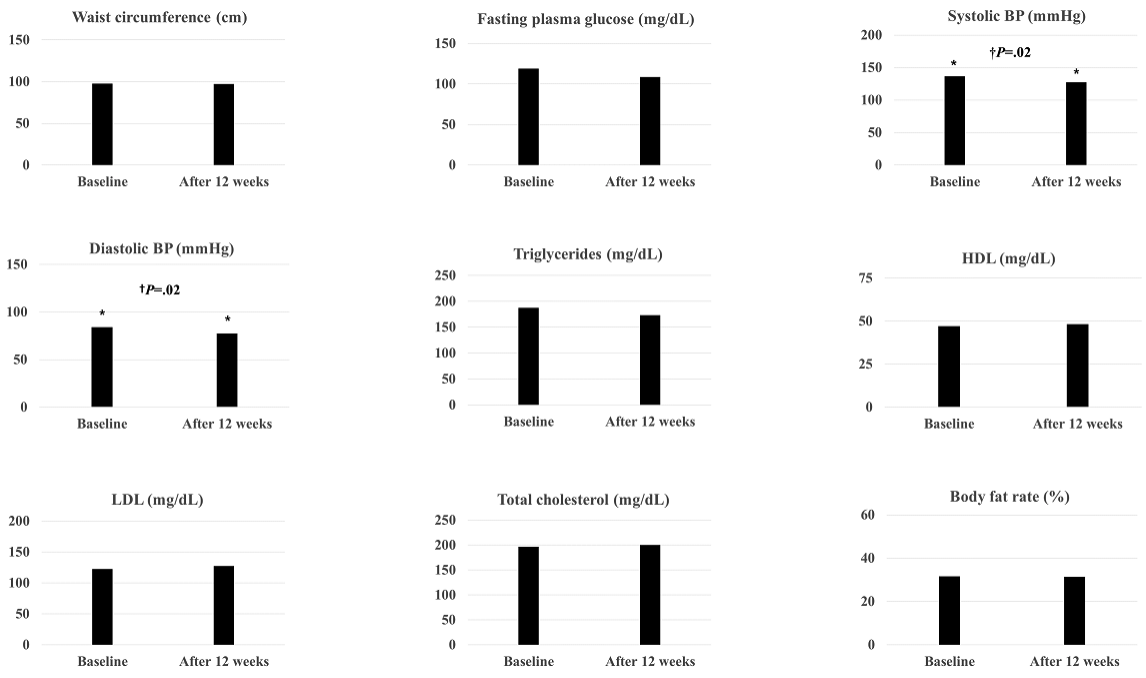
Changes in metabolic components and body compositions after the 12-week intervention. †: Wilcoxon signed-rank test; BP: blood pressure; HDL: high-density lipoprotein; LDL: low-density lipoprotein.

The total number of metabolic impairment components in the 20 participants decreased from 68 to 58, indicating that the mean number of metabolic impairment components per person decreased from 3.4 to 2.9 (Figure 4B). Figure 5 shows the changes in the data of each metabolic component and body composition. After the intervention, systolic BP and diastolic BP significantly decreased by 6.71% and 7.98%, respectively, compared to the baseline assessment (both *P*=.02). The levels of FPG (9.24%) and triglyceride (7.49%) also decreased considerably compared to the baseline; however, these changes were not statistically significant. Furthermore, there were no significant differences in body weight, waist circumference, and body fat between baseline and week 12.

## Discussion

### Principal Results and Comparison With Prior Work

To the best of our knowledge, we conducted the first pilot study to explore the potential and barriers of adoption of connected care technologies using wearable activity trackers with a mobile phone app for metabolic syndrome patients’ management in a clinical setting. Our results showed that this intervention promoted physical activity for 12 weeks, resulting in significant improvement in metabolic impairments—especially in BP—leading to a 45% metabolic syndrome resolution rate. However, we saw a high dropout rate, revealing substantial obstacles in applying this technology in real life.

The main finding of this study was a significant decrease in systolic BP by 9.2 mm Hg (6.71%) and diastolic BP by 6.65 mm Hg (7.98%) 12 weeks after the first use of the device. The degree of reduction in systolic BP observed in our study is equivalent to that obtained through antihypertensive medications with a mean of 8.8 mm Hg (95% confidence interval [CI] −9.58 to −8.02). It was also superior to that obtained through structured exercise interventions with a mean of 4.84 mm Hg (95% CI −5.55 to −4.13) according to a recent meta-analysis of 391 randomized controlled trials comparing the effect of medications and exercise regimens on systolic BP [30]. Additionally, systolic and diastolic BP were the most improved components among the metabolic indexes in our study, which is consistent with prior study, showing that systolic and diastolic BP were most inversely related with steps per day tracked by a pedometer [31]. It is well established that regular walking is effective in BP control by improving vasodilatory function, lowering vasoconstrictor tone, and mainly decreasing endothelin-1 endogenous bioavailability. However, walking is required to be almost daily for a significant improvement in BP [32]. However, many people reportedly find it difficult to maintain daily walking without any motivation or feedback [16]. In this context, the observed step counts and estimated calorie expenditure at weeks 4, 8, and 12 were quite impressive given that more than half the participants had not engaged in any type of exercises at baseline.

For the mean number of steps taken throughout the trial, there was obviously a consistency in the walking behavior among the participants who completed the 12-week intervention. For the first 4 weeks after wearing the device, the participants walked on average 7616 steps daily. This value is equivalent to the “moderately active” level according to the steps-per-day categories and classification system of Tudor-Locke and Bassett [33]. Furthermore, the participants took 17.5% more steps to reach 8244 in the next 4 months. Between weeks 8 and week 12, the number of steps remained at the same level as that shown in the first 4 weeks. Thus, these results indicate that the participants fulfilled the amount of physical activity during the 12 weeks of trial that was recommended in the current guidelines for metabolic syndrome management. Given that only five participants in our study conducted regular exercise at baseline, it was assumed that at least 55% of the participants started being more physically active than before enrollment in the trial.

Although the number of steps that they had walked before enrollment in the study was not assessed, our data showed no significant difference in step counts between those who had regularly exercised before the study and those who had not. It is clinically meaningful for people who were once physically inactive to maintain a moderately active level for 12 weeks, which can be considered a driving contributor to the 45% resolution in metabolic syndrome in our study.

On the other hand, this study failed to see a significant reduction in waist circumference, which is the main indicator of metabolic syndrome. This is in line with the finding of previous studies that saw no improvement in waist circumference or body weight despite having promoted physical activity through feedback using wearable devices [34,35]. The result is possibly due to the increase in physical activity without dietary intervention, which is not sufficient to bring about weight loss.

Traditional metabolic syndrome therapies are focused on reducing insulin resistance through dietary control, physical exercise, and lifestyle modification. However, the majority of the general population have no or difficult access to personally supervised exercise programs or fitness centers with personal health trainers [11]. Moreover, it is challenging for patients with metabolic syndrome to correct their sedentary lifestyle and sustain health-related changes in their everyday lifestyle [21]. Although doctors can prescribe medications, they cannot constantly oversee the routine of their patients. Therefore, it is important to implement strategies to encourage or support physical activity on a daily basis among patients with metabolic syndrome. Mobile phones have evolved as an integral part of people’s lives, and innovative apps support consumers in various ways. With an upsurge in mobile phone distribution, Korea is one of the countries with the highest number of mobile phone users worldwide [36]. The development of mobile phones with advanced computer technology to support Web browsing, third-party apps, wireless connectivity, and sensors (eg, pedometers, accelerometers, and global positioning system trackers) has opened up a new era for personal health self-management [16]. Along with a wide distribution of mobile phones and innovative mobile apps, several low-cost or free health care self-management apps have been developed for continuous and personalized tracking of daily activities. Historically, validated medical devices implanted with sensors have been adopted in clinical studies and targeted research conducted in medical settings. However, technological advances in the measurement of activity (eg, steps), biochemistry (eg, pH), and physiology (eg, blood oxygen saturation) have now fostered the development of patient care and research outside of the hospital setting [37].

Currently, most popular consumer-accessible wearable devices estimate movements via accelerometers that apply algorithms to calculate activity levels (generally in the form of steps taken) and calories expended. These wearable devices differ from traditional pedometers by adding a variety of techniques related to health behavior changes, such as social support or comparisons, goal setting, and rewards [38]. There are various types of pedometers, ranging from simple and inexpensive ones that quantify steps to technologically advanced accelerometers that can measure the amount and intensity of physical activity and total consumed calories in daily life [33-35]. These activity monitors can be worn without major inconvenience, are easy to use, and are compatible with most daily activities [31]. Previous reviews have already reported that physical activity counseling is associated with a significant increase in self-reported daily physical activity levels [39]. However, self-reported measurements of physical activity cannot provide valuable data because they are inaccurate, and there may be a social desirability bias [31].

Studies have found that interventions consisting of counseling combined with activity monitoring have a positive effect on daily physical activity levels in participants with chronic disease [7,9-11]. However, information regarding the effects of counseling with activity monitoring on objective measures of physical activity and health-related outcomes (eg, BMI, BP, and lipid profiles) remains limited to date. Although the use of wearable devices, such as wristbands, smart watches, and biomonitors, has considerably increased worldwide, comparatively few studies have investigated their effect on the resolution of metabolic syndrome. The most important limitation of wearable devices is that for the majority of users, most devices fail to drive long-term sustained engagement. A recent Pew Internet and American Life survey showed that over 59% of customers in the United States who own an activity tracker no longer use it and one-third of customers who own a device no longer use it after 6 months [40]. Designing a strategy to ensure sustained engagement is the key to the long-term success of wearable devices in health care. Most products and services offering a range of uses fail to cause a meaningful change in users’ health-related behaviors and habits; for example, activity tracker users rapidly abandon devices that do not help them to make positive changes and, ultimately, they fail to achieve self-care in health [41,42]

Our study observed a significant dropout rate that was higher than expected. One of the possible reasons was that our participants were relatively older (median age 46 years) compared to previous studies [16,36]. The participants who failed to complete the trial were older than those who completed it. This is consistent with previous research that investigated the potential sociodemographic characteristics of individuals using wearable activity trackers or mobile phone apps for physical activity surveillance [38]. We included participants who were daily mobile phone users of more than 3 months to minimize the dropout rate caused by unfamiliarity with handling of the app or of the Bluetooth on mobile phones required for connecting the wearable device. Relevant research showed that the older participants were more likely to be unwilling to try to address the issues with mobile phones when that happens [41]. This can be an explanation for why most of those who dropped out in our study did so during the first 4 weeks after they started using the device. Despite efforts of researchers to solve the technical issues when they happened and encourage the users to try again, the majority of participants who experienced the problems withdrew their consent due to dissatisfaction with the device. Particularly, older participants had trouble learning how to operate the device or getting used to it. Additional education should have been provided for participants aged 50 years and older to resolve any technical problems. This phenomenon illustrates the importance of making smart medical devices user-friendly in clinical settings, and suggests the main point to be addressed when health providers consider applying this digital health self-tracking system to their patients. Devices that incorporate a function allowing users to change their habits will encourage sustained behavioral changes and result in long-term self-management of health care. Further study is needed to identify factors that influence users’ intention to continue using connected care technologies and the reasons for usage discontinuance.

### Limitations

This analysis has several limitations that warrant consideration when interpreting the results. First, the sample size was small, and the intervention period was relatively short. The participants were individuals undergoing health examination in Korea; therefore, it is not possible to generalize our findings to other ethnicities or geographic regions. Secondly, a significant proportion of the participants were lost to follow-up. Moreover, this study included only a few middle-aged women due to the low number of women volunteers. During the study, we were unaware if any participant received another commercialized program for dietary control. Finally, our study had no control group in which participants were provided with educational programs to increase their physical activities instead of using the wearable device. As a result, the positive findings seen in this study might have come from participants’ behavior changes such as healthy dietary choice or motivation by the nurse’s telephone feedback rather than the wearable device itself.

Despite these limitations, our study is considerably valuable owing to several strengths. Firstly, to the best of the authors’ knowledge, this is the first study to explore the potential and possible barriers to the adaptation of a wearable device connected to a mobile phone app for the management of metabolic syndrome in a clinical setting. Another primary strength of this study is the comprehensive health examination-based screening performed that resulted in an accurate diagnosis of metabolic syndrome by a family medicine doctor, unlike previous studies in which the diagnosis of metabolic syndrome was based on self-reports. Therefore, recall bias and the risk of disease misclassification were minimized. Furthermore, the study collated detailed information on participants’ medical history, demographics, and laboratory results, which allowed the exclusion of ineligible participants and adjustments for important potential confounders during the analysis.

### Conclusion

In conclusion, this study found that a 12-week intervention via feedback based on a wearable physical activity monitor helped metabolic syndrome patients to engage in more regular walking and improved impaired metabolic components especially in terms of BP. However, some practical challenges need to be addressed with respect to patients’ adherence and sustained engagement.

## Appendix

Multimedia Appendix 1 Content of the telephone feedback by a trained nurse.

## Abbreviations

ANOVA: analysis of variance
BMI: body mass index
BP: blood pressure
FPG: fasting plasma glucose
HDL-C: high-density lipoprotein cholesterol
IQR: interquartile range
TG: triglyceride

## References

[1] Eckel RH, Grundy SM, Zimmet PZ (2005). The metabolic syndrome. Lancet.

[2] Wilson PW, D'Agostino RB, Parise H, Sullivan L, Meigs JB (2005). Metabolic syndrome as a precursor of cardiovascular disease and type 2 diabetes mellitus. Circulation.

[3] Sattelmair J, Pertman J, Ding EL, Kohl HW, Haskell W, Lee I (2011). Dose response between physical activity and risk of coronary heart disease: a meta-analysis. Circulation.

[4] Bird SR, Hawley JA (2016). Update on the effects of physical activity on insulin sensitivity in humans. BMJ Open Sport Exerc Med.

[5] Woodcock J, Franco OH, Orsini N, Roberts I (2011). Non-vigorous physical activity and all-cause mortality: systematic review and meta-analysis of cohort studies. Int J Epidemiol.

[6] Lee I, Shiroma EJ, Lobelo F, Puska P, Blair SN, Katzmarzyk PT, Lancet Physical Activity Series Working Group (2012). Effect of physical inactivity on major non-communicable diseases worldwide: an analysis of burden of disease and life expectancy. Lancet.

[7] Chiauzzi E, Rodarte C, DasMahapatra P (2015). Patient-centered activity monitoring in the self-management of chronic health conditions. BMC Med.

[8] Takemoto M, Lewars B, Hurst S, Crist K, Nebeker C, Madanat H, Nichols J, Rosenberg DE, Kerr J (2018). Participants' perceptions on the use of wearable devices to reduce sitting time: qualitative analysis. JMIR Mhealth Uhealth.

[9] Bavan L, Surmacz K, Beard D, Mellon S, Rees J (2019). Adherence monitoring of rehabilitation exercise with inertial sensors: a clinical validation study. Gait Posture.

[10] Fernández Peruchena CM, Prado-Velasco M (2010). Smart sensors and virtual physiology human approach as a basis of personalized therapies in diabetes mellitus. Open Biomed Eng J.

[11] Vaes A, Cheung A, Atakhorrami M, Groenen M, Amft O, Franssen F, Wouters E, Spruit M (2013). Effect of 'activity monitor-based' counseling on physical activity and health-related outcomes in patients with chronic diseases: A systematic review and meta-analysis. Ann Med.

[12] Umpierre D, Ribeiro PA, Kramer CK, Leitão CB, Zucatti AT, Azevedo MJ, Gross JL, Ribeiro JP, Schaan BD (2011). Physical activity advice only or structured exercise training and association with HbA1c levels in type 2 diabetes: a systematic review and meta-analysis. JAMA.

[13] Quinn CC, Butler EC, Swasey KK, Shardell MD, Terrin MD, Barr EA, Gruber-Baldini AL (2018). Mobile diabetes intervention study of patient engagement and impact on blood glucose: mixed methods analysis. JMIR Mhealth Uhealth.

[14] Alharbi M, Straiton N, Gallagher R (2017). Harnessing the potential of wearable activity trackers for heart failure self-care. Curr Heart Fail Rep.

[15] Robinson SA, Shimada SL, Quigley KS, Moy ML (2019). A web-based physical activity intervention benefits persons with low self-efficacy in COPD: results from a randomized controlled trial. J Behav Med.

[16] Bort-Roig J, Gilson ND, Puig-Ribera A, Contreras RS, Trost SG (2014). Measuring and influencing physical activity with smartphone technology: a systematic review. Sports Med.

[17] Wang J, Cai C, Padhye N, Orlander P, Zare M (2018). A behavioral lifestyle intervention enhanced with multiple-behavior self-monitoring using mobile and connected tools for underserved individuals with type 2 diabetes and comorbid overweight or obesity: pilot comparative effectiveness trial. JMIR Mhealth Uhealth.

[18] Turner-McGrievy GM, Beets MW, Moore JB, Kaczynski AT, Barr-Anderson DJ, Tate DF (2013). Comparison of traditional versus mobile app self-monitoring of physical activity and dietary intake among overweight adults participating in an mHealth weight loss program. J Am Med Inform Assoc.

[19] Lyons EJ, Swartz MC, Lewis ZH, Martinez E, Jennings K (2017). Feasibility and acceptability of a wearable technology physical activity intervention with telephone counseling for mid-aged and older adults: a randomized controlled pilot trial. JMIR Mhealth Uhealth.

[20] van Dantzig S, Geleijnse G, van Halteren At (2012). Toward a persuasive mobile application to reduce sedentary behavior. Pers Ubiquit Comput.

[21] Alberti KG, Eckel RH, Grundy SM, Zimmet PZ, Cleeman JI, Donato KA, Fruchart J, James WPT, Loria CM, Smith SC, International Diabetes Federation Task Force on EpidemiologyPrevention, Hational Heart‚ Lung‚Blood Institute, American Heart Association, World Heart Federation, International Atherosclerosis Society, International Association for the Study of Obesity (2009). Harmonizing the metabolic syndrome: a joint interim statement of the International Diabetes Federation Task Force on Epidemiology and Prevention; National Heart, Lung, and Blood Institute; American Heart Association; World Heart Federation; International Atherosclerosis Society; and International Association for the Study of Obesity. Circulation.

[22] Lee SY, Park HS, Kim DJ, Han JH, Kim SM, Cho GJ, Kim DY, Kwon HS, Kim SR, Lee CB, Oh SJ, Park CY, Yoo HJ (2007). Appropriate waist circumference cutoff points for central obesity in Korean adults. Diabetes Res Clin Pract.

[23] Corrao G, Bagnardi V, Zambon A, La Vecchia C (2004). A meta-analysis of alcohol consumption and the risk of 15 diseases. Prev Med.

[24] Johnson JL, Slentz CA, Houmard JA, Samsa GP, Duscha BD, Aiken LB, McCartney JS, Tanner CJ, Kraus WE (2007). Exercise training amount and intensity effects on metabolic syndrome (from Studies of a Targeted Risk Reduction Intervention through Defined Exercise). Am J Cardiol.

[25] Bassett DJ, Toth L, LaMunion S, Crouter S (2017). Step counting: a review of measurement considerations and health-related applications. Sports Med.

[26] Tudor-Locke C, Barreira T, Schuna J (2015). Comparison of step outputs for waist and wrist accelerometer attachment sites. Med Sci Sports Exerc.

[27] Troiano R, McClain J, Brychta R, Chen K (2014). Evolution of accelerometer methods for physical activity research. Br J Sports Med.

[28] Tudor-Locke C, Bassett D, Shipe M, McClain J (2011). Pedometry methods for assessing free-living adults. J Phys Act Health.

[29] Turner-McGrievy G, Tate D (2011). Tweets, apps, and pods: results of the 6-month Mobile Pounds Off Digitally (Mobile POD) randomized weight-loss intervention among adults. J Med Internet Res.

[30] Naci H, Salcher-Konrad M, Dias S, Blum MR, Sahoo SA, Nunan D, Ioannidis JP (2018). How does exercise treatment compare with antihypertensive medications? A network meta-analysis of 391 randomised controlled trials assessing exercise and medication effects on systolic blood pressure. Br J Sports Med.

[31] Jahan N, Shenoy S (2017). Relation of pedometer steps count & self reported physical activity with health indices in middle aged adults. Diabetes Metab Syndr.

[32] Sabbahi A, Arena R, Elokda A, Phillips S (2016). Exercise and hypertension: uncovering the mechanisms of vascular control. Prog Cardiovasc Dis.

[33] Tudor-Locke C, Bassett D (2004). How many steps/day are enough? Preliminary pedometer indices for public health. Sports Med.

[34] Pal S, Cheng C, Egger G, Binns C, Donovan R (2009). Using pedometers to increase physical activity in overweight and obese women: a pilot study. BMC Public Health.

[35] Yang Y, Wang C, Wang J, Lin C, Yang Y, Wang J, Yang Y, Yang Y (2017). The Effects of an Activity Promotion System on active living in overweight subjects with metabolic abnormalities. Obes Res Clin Pract.

[36] Jee H (2017). Review of researches on smartphone applications for physical activity promotion in healthy adults. J Exerc Rehabil.

[37] Sarasohn-Kahn J (2013). California Healthcare Foundation.

[38] Lyons EJ, Lewis ZH, Mayrsohn BG, Rowland JL (2014). Behavior change techniques implemented in electronic lifestyle activity monitors: a systematic content analysis. J Med Internet Res.

[39] Orrow G, Kinmonth A, Sanderson S, Sutton S (2012). Effectiveness of physical activity promotion based in primary care: systematic review and meta-analysis of randomised controlled trials. BMJ.

[40] Fox S, Duggan M (2013). Pew Research Center.

[41] Paré G, Leaver C, Bourget C (2018). Diffusion of the digital health self-tracking movement in Canada: results of a national survey. J Med Internet Res.

[42] Strain T, Wijndaele K, Brage S (2019). Physical activity surveillance through smartphone apps and wearable trackers: examining the UK potential for nationally representative sampling. JMIR Mhealth Uhealth.

