# Supplementary material for: Feedback on Physical Activity Through a Wearable Device Connected to a Mobile Phone App in Patients With Metabolic Syndrome: Pilot Study
Source: JMIR Mhealth Uhealth. 2019 Jun 18;7(6):e13381. doi: 10.2196/13381 (PMC6604502; doi:10.2196/13381)
Supplement: Multimedia Appendix 1 [file mhealth_v7i6e13381_app1.docx]

Mutlimedia Appendix 1. Content of the telephone feedback by a trained nurse

| Start with greeting and confirm the identification of the subject  Let the subject talk about the experiences of using the device and their goal-setting  Inform them of how many steps they have taken for the last two weeks and how many calories they have consumed by workouts.  Remind them of their goal of physical activity  If they failed to achieve the goal, ask them if there is any obstacle that stops them from performing physical activity.  If the problem they face sounds associated with their sedentary lifestyle, give some practical advice to overcome this, raise awareness on the risks of being physical inactive, and enhance knowledge of the benefits of physical activity.  Encourage them to engage in more physical activity.  If they reached the goal, give some complementary comments and tell them to keep up the good work.  See if there is any technical issue with the device or application that discourages from using them  If the problem needs fixing by a technician, ask the user to bring the device and smartphone to have them fixed as soon as possible.  Wrap up the conversation with a schedule for the next phone call. |
| --- |
